# Intravenous Colistin in the treatment of multidrug-resistant Acinetobacter in neonates

**DOI:** 10.1186/s12941-016-0126-4

**Published:** 2016-02-12

**Authors:** Manar Al-lawama, Haytham Aljbour, Asma Tanash, Eman Badran

**Affiliations:** The University of Jordan, Amman, Jordan; Jordan University Hospital, Amman, Jordan

**Keywords:** Colistin, Sepsis, Neonate, Multidrug-resistant Acinetobacter, Eosinophilia

## Abstract

**Background:**

Neonatal sepsis caused by multidrug-resistant gram-negative bacteria has been reported in different parts of the world. It is a major threat to neonatal care, carrying a high rate of morbidity and mortality. While Colistin is the treatment of choice, few studies have reported its use in neonatal patients.

**Methods:**

A retrospective descriptive study of all neonatal patients who had multidrug-resistant Acinetobacter sepsis and were treated with Colistin over a 2-year period. Patients’ charts and hospital laboratory data were reviewed.

**Results:**

During the study period, 21 newborns were treated with Colistin. All had sepsis evident by positive blood culture and clinical signs of sepsis. The median gestational age and birth weight were 33 weeks (26–39) and 1700 g (700–3600), respectively. Nine (43 %) were very low birth weight infants. Eighteen (86 %) were preterm infants. Nineteen (91 %) newborns survived. No renal impairment is documented in any of our patients. Fourteen (67 %) of our patients had elevated eosinophil counts following Colistin treatment, for those patients, the average eosinophilic counts ± standard deviation before and after Colistin therapy were 149.08 ± 190.38 to 1193 ± 523.29, respectively, with a p value of less than 0.0001.

**Conclusion:**

Our study showed that Colistin was both effective and safe for treating multidrug-resistant Acinetobacter neonatal sepsis. This is a retrospective study. No universal protocol was used for the patients. The factors that might affect the response or cause side effects are difficult to evaluate.

## Background

Neonatal sepsis caused by multidrug-resistant gram-negative bacteria has been reported in different parts of the world [1–5]. It is a major threat to neonatal care, carrying a high rate of morbidity and mortality [6–9]. While Colistin is the treatment of choice, [10, 11] few studies have reported its use in neonatal patients [12–14]. The treatment protocol, safety and efficacy have not yet been established.

In this study, we will report our unit’s experience on the use of Colistin to treat neonatal gram-negative sepsis caused by multidrug-resistant Acinetobacter. We describe the treatment protocol and report the side effects and response to therapy.

## Methods

The design was a retrospective descriptive study of all neonatal patients who had multidrug-resistant Acinetobacter sepsis and were treated with Colistin over a 2-year period. It was conducted at a level 3 neonatal unit in Amman - Jordan that had a capacity of 30 beds.

This paper is part of the neonatal sepsis study and was approved by the deanship of scientific research.

All neonates with blood culture-proven sepsis from multidrug-resistant Acinetobacter were identified. Those treated with Colistin were included.

Patients’ charts and hospital laboratory data were reviewed. Patients’ demographics, culture results, antimicrobial sensitivity patterns, laboratory investigation results, Colistin dose, frequency, duration of treatment, co-administered antibiotics, follow-up blood culture results and mortality were recorded.

Sepsis is defined as a positive blood culture with the presence of clinical signs of sepsis. Late-onset sepsis is defined as sepsis occurring after 72 h of life.

The indications for Colistin were the following: documentation of Carbapenems-resistant gram-negative bacteria in the blood culture or empirically when newborns present with septic shock during an outbreak of multidrug-resistant gram-negative sepsis.

Response to treatment was divided into clinical and microbiological response. The clinical response was defined as resolution of symptoms and survival. The microbiological response was defined as a negative blood culture post-Colistin therapy.

Central line-associated bloodstream infection (CLABSI) is defined as sepsis with a central line in situ or if the central line was present up to 48 h before the date of a positive blood culture.

SPSS^®^ version 21 (SPSS Inc., Chicago, IL, USA) was used to conduct statistical analyses. *p* values <0.05 were considered statistically significant.

## Results

During the study period, 21 newborns were treated with Colistin. All had sepsis evident by positive blood culture and clinical signs of sepsis.

All septic episodes were caused by Acinetobacter species. Nineteen (90 %) were Carbapenems resistant. All were sensitive to Colistin.

The median gestational age and birth weight were 33 weeks (26–39) and 1700 g (700–3600), respectively. Nine (43 %) were very low birth weight infants. Eighteen (86 %) were preterm infants. The characteristics of the newborn patients who received intravenous Colistin are presented in Table 1.

**Table 1:** Clinical characteristics of neonates with multidrug-resistant Acinetobacter sepsis who were treated with Colistin

Preterm <37 weeks	18 (86 %)
Gestational age—median (range)	33 (26–40)
Birth weight—mean (range)	1700 (800–3600)
VLBW	9 (43 %)
Male gender	13 (62 %)
Pre-eclampsia	3 (14 %)
PROM	3 (14 %)
Cesarean section	16 (76 %)
Apgar scores- mean (range) (min)	
1	7 (3–8)
5	9 (7–9)
CLABSI number (%)	14 (67 %)
Late sepsis number (%)	21 (100 %)
Mean age at sepsis (days)	10 (4–30)
Carpapenems resistance (%)	19 (90 %)
Day of sepsis Colistin started	2.5 (1–6)
Treatment duration (days)	17(10–21)
Dose U/kg/day	70,000 (30,000–75,000)
Day of microbiological clearance—mean (range)	3 (1–8)
Mortality (%)	2 (9.5 %)

*VLBW* very low birth weight, *PROM* prolonged rupture of membranes, *CLABSI* central line associated blood stream infection

Intravenous Colistin was started for late-onset sepsis in all patients, with median post-natal age of 10 days (4–30). The average duration from presentation to the start of Colistin therapy was 2.5 (1–6) days. The average daily dose was 70,000 IU/kg/D (30,000–75,000). The average duration of treatment was 17 days (10–21).

Nineteen (91 %) newborns survived. Nineteen patients had follow-up blood culture to document clearance. All had negative cultures with an average duration for clearance of 3 days (range 1–8). One patient continued to have positive culture until day 8 of treatment.

Colistin was administered, with other medications at some time during the treatment for all patients (Table 2). With respect to coverage for gram-negative bacteria, none of our patients had Colistin as monotherapy. Eighteen (86 %) received Colistin with Imipenem and Amikacin/Gentamicin for the entire duration of treatment. One patient received Colistin with Amikacin for the entire duration of treatment. One patient received Colistin with Imipenem for the entire treatment duration, and one patient was treated with Imipenem and Amikacin for the first week of treatment; then, the patient received Colistin alone for the second week.

**Table 2:** Concomitant antibiotics and antifungal treatment during Colistin administration

Drug	Number (%)
Vancomycin	9 (43)
Imipinem	19 (90)
Amikacin	17 (81)
Fluconazole	5 (24)
Amphotericin	3 (14)
Gentamicin	2 (9.5)

No renal impairment is documented in any of our patients. The mean creatinine and standard deviation for our patients during pre- and post-Colistin administration were 0.47 (0.7) and 0.27 (0.35) mg/dL, respectively (p value 0.249). None of the patients had a post-Colistin creatinine elevation of more than 0.5 mg/dL.

**Fig. 1:**
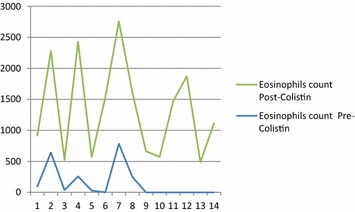
Eosinophils count pre-Colistin and Post Colistin treatment in 14 patients

Three patients developed one episode of hypokalemia, two had one episode of hypocalcaemia, and two had hyponatremia; only nine patients had their magnesium levels measured during treatment, and two of them had hypomagnesaemia. Fourteen (67 %) of our patients had elevated eosinophil counts following Colistin treatment, for those patients, the average eosinophilic counts ± standard deviation before and after Colistin therapy were 149.08 ± 190.38 to 1193 ± 523.29, respectively, with a p value of less than 0.0001 (Fig. 1).

## Discussion

Our study showed that Colistin was both effective and safe for treating multidrug-resistant Acinetobacter neonatal sepsis.

Twenty-one newborn infants received Colistin and had sepsis demonstrated by blood culture. All cases had Acinetobacter species, and 19 were Carbapenems-resistant Acinetobacter species.

Thus far, this is the only study in which all newborns had Acinetobacter-proven sepsis by blood culture. Twenty-one preterm infants treated with Colistin were reported by Alan et al., and only nine of those had a positive blood culture [12]. In a report by Jajoo et al., only 13 newborns had Acinetobacter isolated from blood samples [13]. Isolation of certain pathogens from places other than the blood might reflect colonization. In addition, newborns who were treated empirically might have causes for their sepsis beyond multidrug-resistant gram-negative bacteria, and they might not have bacterial sepsis. Our study reports the largest series of newborns with sepsis from multidrug-resistant Acinetobacter according to blood culture who were treated with Colistin.

We did not use a loading dose in our protocol, which is similar to previous reports [12–14]. The average dose was 70,000 IU/kg/day, and most of our patients received 75,000 IU/kg/day. This dose is higher than previously reported doses [12–14]. The lowest dose was 30,000 IU/kg/D, and the baby who received this dose survived with microbiological clearance after 2 days of treatment.

In our population, the clinical efficacy was 91 %. Nineteen newborns had follow-up blood cultures. Microbiological clearance was documented in all of them. The average time for negative culture was 3 days. Two newborn infants had clearance documented after 8 days of treatment; one of them did not have follow-up cultures until day 8 and had a stable course, while the other had cultures drawn every 2 days, all of which grew Acinetobacter until day 8. Unfortunately, this baby died. During the study period, there was no protocol for repeating blood cultures, which might contribute to the variable time to clearance.

The survival rate reported in our study is higher than previous reports [12, 13]. This might be from the higher Colistin dose than previous reports [12, 13]. Additionally, the day for when Colistin treatment was initiated for sepsis was not reported in other studies. Our babies might have been started on treatment earlier.

Nephrotoxicity is the most commonly reported adverse effect of Colistin therapy [15, 16]. Previous studies on the neonatal population have reported acute kidney injury and electrolyte disturbances [12, 13]. In our series of patients, there was no evidence of acute kidney injury and the electrolyte imbalances were insignificant.

Fourteen (67 %) patients in our study had an elevated eosinophilic count following Colistin treatment. Because the treatment team did not note this during treatment, no clinical correlation was made between the increase in eosinophil count and symptoms. Additionally, few patients had a follow-up CBC after the end of treatment. As a result, recovery to normal count could not be documented. An allergic reaction to Colistin was previously reported, which varies between mild itching and episodes of rash to cough, sore throat and bronchoconstriction [17–19]. Only two reports of eosinophilia were found. In an old study, two adult patients developed eosinophilia [20]. Recently there was another report of an elderly lady who developed hypersensitivity pneumonitis [21]. This is the first report on eosinophilia with Colistin treatment in pediatric and neonatal patients. A prospective study is needed to evaluate the clinical implications of such a side effect.

Our study provides physician taking care of neonatal patients with valuable information regarding the treatment of multidrug resistant Acinetobacter species with Colistin. This is a retrospective study. No universal protocol was used for the patients. The factors that might affect the response or cause side effects are difficult to evaluate.
